# Supplementary material for: Complete classification of steerability under local filters and its relation with measurement incompatibility
Source: Nat Commun. 2022 Aug 25;13:4973. doi: 10.1038/s41467-022-32466-y (PMC9411635; doi:10.1038/s41467-022-32466-y)
Supplement: Supplementary file 1 — Supplementary Information [file 41467_2022_32466_MOESM1_ESM.pdf]

Supplemental Information: Complete classification of steerability under local filters and its relation with measurement incompatibility

Huan-Yu Ku[1,2,3], Chung-Yun Hsieh[4,*], Shin-Liang Chen[1,5,6,+], Yueh-Nan Chen[1,†], and Costantino Budroni[2,3]

[1]Department of Physics and Center for Quantum Frontiers of Research & Technology (QFort), National Cheng Kung University, Tainan 701, Taiwan
[2]Faculty of Physics, University of Vienna, Boltzmanngasse 5, 1090 Vienna, Austria
[3]Institute for Quantum Optics and Quantum Information (IQOQI), Austrian Academy of Sciences, Boltzmanngasse 3, 1090 Vienna, Austria
[4]ICFO – Institut de Ciéncies Fotóniques, The Barcelona Institute of Science and Technology, Castelldefels 08860, Spain
[5]Dahlem Center for Complex Quantum Systems, Freie Universität Berlin, 14195 Berlin, Germany
[6]Department of Physics, National Chung Hsing University, Taichung 40227, Taiwan
[*]andrew791006@gmail.com
[+]shin.liang.chen@email.nchu.edu.tw
[†]yuehnan@mail.ncku.edu.tw

ABSTRACT

In this supplemental information we provide a brief summary of the free operations in the resource theory of steering, a detailed proof of Theorem 3 from the main text, an example of a non-maximally entangled state that provides maximal steerability, the explicit calculation of the conversion rates, and an example with a qubit assemblage.

1 SUPPLEMENTARY NOTE 1: Free Operations in the Resource Theory of Quantum Steering

In this section, we briefly recall the definition of free operations in the resource theory of steering[1]. These are defined as the one-way (1W) stochastic (S) local operations and classical communications (LOCCs), or 1W-SLOCC operations. They can be briefly described as follows[1]. First, Bob performs a measurement on his local system and obtains an outcome ω (local filtering operation), represented by Kraus operators $\{K_\omega\}$. Then, he communicates the output to Alice, who applies some local classical pre and postprocessing maps (local wirings) that depends on the outcome ω. In summary, the transformed assemblage can be computed as

$$\sigma^{\omega}_{a'|x'} = \frac{1}{p(\omega)} \sum_{a,x} p(x|x'\omega) p(a'|a,x,x',\omega) K_\omega \sigma_{a|x} K_\omega^\dagger, \tag{1}$$

where

$$p(\omega) = \sum_a \mathrm{tr}[K_\omega \sigma_{a|x} K_\omega^\dagger] \tag{2}$$

is the probability of postselecting on the outcome ω, sometimes also denoted as $p_{\rm succ}$, i.e., the probability of successfully filtering the outcome ω. Without loss of generality, note that one can consider the case of a single Kraus operator for each outcome ω. In fact, the case of multiple Kraus operators for each outcome can be absorbed by a proper postprocessing on Alice's side, namely a coarse-graining of the outcomes ω (see also the discussion in Ref. [2]). We denote the set of local filtering with one Kraus operator as LF_1. Operations in LF_1 correspond to the subset of 1W-SLOCC operations in which Alice does not apply any postprocessing but only one of the outcomes of the measurement, e.g., $\omega = 0$, is selected. This corresponds to substituting in Eq. (38) of the main text$p(x|x',\omega) = \delta_{x,x'}\delta_{\omega,0}$ and $p(a'|a,x,x',\omega) = \delta_{a,a'}\delta_{\omega,0}$.

2 SUPPLEMENTARY NOTE 2: Proof of Theorem 3

The first step is to again use the property in Eq. (36) of the main text,as shown in Ref. [3], but this time with the maximally entangled state $|\psi\rangle = \sum_i |ii\rangle/\sqrt{d}$. We have

$$(\sigma^{\boldsymbol{A}}_{a|x})^T := \frac{A^T_{a|x}}{d} = \left[A_{a|x} \otimes \mathbb{I}|\psi\rangle\langle\psi|\right]. \tag{3}$$

Notice that an extra transposition appears in $A_{a|x}$ here. This transposition, however, is irrelevant at the level of the calculation of the robustness, both in terms of steering and incompatibility. In fact, given a state assemblage $\boldsymbol{\sigma}$ and a measurement assemblage $\boldsymbol{A}$, we have $\mathrm{SR}(\boldsymbol{\sigma}) = \mathrm{SR}(\boldsymbol{\sigma}^{\mathrm{T}})$ and $\mathrm{IR}(\boldsymbol{A}) = \mathrm{IR}(\boldsymbol{A}^{\mathrm{T}})$, respectively. Theorem 2 can now be applied to find the assemblage that maximizes the robustness within the same class of $\sigma^{\boldsymbol{A}}_{a|x}$. Following the proof of Theorem 2, we construct the operator η_ε and the corresponding assemblage $\boldsymbol{\sigma}^\varepsilon$ that approximates the incompatibility robustness, i.e.,

$$\sigma^\varepsilon_{a|x} = d\eta_\varepsilon^{1/2}(\sigma^{\boldsymbol{A}}_{a|x})^T\eta_\varepsilon^{1/2} = \eta_\varepsilon^{1/2}A^T_{a|x}\eta_\varepsilon^{1/2}, \tag{4}$$

giving $\mathrm{SR}(\boldsymbol{\sigma}^\varepsilon) \geq \mathrm{IR}(\boldsymbol{A}) - \varepsilon$. We now define the optimal state

$$\rho^\varepsilon_{\mathrm{AB}} := d[(\mathbb{I}\otimes\eta_\varepsilon^{1/2})|\psi\rangle\langle\psi|(\mathbb{I}\otimes\eta_\varepsilon^{1/2})], \tag{5}$$

and show that $\boldsymbol{\sigma}^\varepsilon$ arises from the measurement assemblage $\boldsymbol{A}$ on it. We have

$$\begin{aligned}
&\mathrm{tr}_{\mathrm{A}}[A_{a|x}\otimes\mathbb{I}\rho^\varepsilon_{\mathrm{AB}}]\\
&= \mathrm{tr}_{\mathrm{A}}[A_{a|x}\otimes\mathbb{I}d((\mathbb{I}\otimes\eta_\varepsilon^{1/2})|\psi\rangle\langle\psi|(\mathbb{I}\otimes\eta_\varepsilon^{1/2}))]\\
&= \mathrm{tr}_{\mathrm{A}}[(A_{a|x}\otimes\mathbb{I})^T d(\mathbb{I}\otimes\eta_\varepsilon^{1/2})|\psi\rangle\langle\psi|(\mathbb{I}\otimes\eta_\varepsilon^{1/2})]\\
&= d\eta_\varepsilon^{1/2}\mathrm{tr}_{\mathrm{A}}[(A_{a|x}\otimes\mathbb{I})|\psi\rangle\langle\psi|]\eta_\varepsilon^{1/2}\\
&= \eta_\varepsilon^{1/2}{A_{a|x}}^T\eta_\varepsilon^{1/2} = \sigma^\varepsilon_{a|x}.
\end{aligned} \tag{6}$$

To conclude, it is sufficient to show that $\rho^\varepsilon_{\mathrm{AB}}$ is a state. Clearly, it is positive by construction, and the normalization is

$$\begin{aligned}
\mathrm{tr}[\rho^\varepsilon_{\mathrm{AB}}] &= \mathrm{tr}[d((\mathbb{I}\otimes\eta_\varepsilon^{1/2})|\psi\rangle\langle\psi|(\mathbb{I}\otimes\eta_\varepsilon^{1/2}))]\\
&= \mathrm{tr}[d(\mathbb{I}\otimes\eta_\varepsilon)|\psi\rangle\langle\psi|] = \mathrm{tr}[\mathbb{I}\eta_\varepsilon] = 1,
\end{aligned} \tag{7}$$

where we use the property of the maximally entangled state $d\mathrm{tr}[A\otimes B|\psi\rangle\langle\psi|] = \mathrm{tr}[A^TB]$.

3 SUPPLEMENTARY NOTE 3: Example: non-Maximally Entangled State Provides Maximal Steerability

Many examples can be generated to achieve our goal by considering two random projectors in a high dimensional system. In the following, we present a concrete example with Alice's measurement assemblage $\boldsymbol{A}$:

$$A_{1|1} = \begin{pmatrix} 0.0055 & -0.0007+0.0469i & -0.0257+0.0416i & 0.0048+0.0285i\\ -0.0007-0.0469i & 0.4033 & 0.3610+0.2153i & 0.2445-0.0445i\\ -0.0257-0.0416i & 0.3610-0.2153i & 0.4381 & 0.1951-0.1703i\\ 0.0048-0.0285i & 0.2445+0.0445i & 0.1951+0.1703i & 0.1531 \end{pmatrix}$$

$$A_{2|1} = \begin{pmatrix} 0.9945 & 0.0007-0.0469i & 0.0257-0.0416i & -0.0048-0.0285i\\ 0.0007+0.0469i & 0.5967 & -0.3610-0.2153i & -0.2445+0.0445i\\ 0.0257+0.0416i & -0.3610+0.2153i & 0.5619 & -0.1951+0.1703i\\ -0.0048+0.0285i & -0.2445-0.0445i & -0.1951-0.1703i & 0.8469 \end{pmatrix}$$

$$A_{1|2} = \begin{pmatrix} 0.2905 & 0.3268-0.1672i & 0.0664+0.1328i & -0.0528+0.2157i\\ 0.3268+0.1672i & 0.4638 & -0.0018+0.1876i & -0.1835+0.2123i\\ 0.0664-0.1328i & -0.0018-0.1876i & 0.0759 & 0.0865+0.0734i\\ -0.0528-0.2157i & -0.1835-0.2123i & 0.0865-0.0734i & 0.1698 \end{pmatrix}$$

$$A_{2|2}=\begin{pmatrix} 0.7095 & -0.3268+0.1672i & -0.0664-0.1328i & 0.0528-0.2157i \\ -0.3268-0.1672i & 0.5362 & 0.0018-0.1876i & 0.1835-0.2123i \\ -0.0664+0.1328i & 0.0018+0.1876i & 0.9241 & -0.0865-0.0734i \\ 0.0528+0.2157i & 0.1835+0.2123i & -0.0865+0.0734i & 0.8302 \end{pmatrix}.$$

In this case, the corresponding incompatibility robustness is $\mathrm{IR}(\boldsymbol{A})=0.1481$. When we consider the shared state to be the maximally entangled state $|\psi\rangle=\sum_{i=0}^{3}\frac{1}{\sqrt{4}}|i\rangle\otimes|i\rangle$, the value of the steering robustness with the corresponding state assemblage is $\mathrm{SR}(\boldsymbol{A}^{\mathrm{T}}/4)=0.0740$. The numerical solution of $\mathrm{IR}(\boldsymbol{A})$ provides the optimal reduced state:

$$\eta=\begin{pmatrix} 0.1882+0.0000i & 0.0975-0.1104i & -0.0520+0.0359i & -0.0876+0.1591i \\ 0.0975+0.1104i & 0.3499+0.0000i & 0.1490+0.0873i & -0.0298-0.0108i \\ -0.0520-0.0359i & 0.1490-0.0873i & 0.2285+0.0000i & 0.1284-0.1084i \\ -0.0876-0.1591i & -0.0298+0.0108i & 0.1284+0.1084i & 0.2334+0.0000i \end{pmatrix}.$$

We now apply Eq. (42) of the main text to this measurement assemblage. Therefore, we can generate the bipartite state such that the steering robustness of the generating assemblage $\boldsymbol{\sigma}^{\eta}$ is associated with the incompatibility of the measurement assemblage $\boldsymbol{A}$, namely $\mathrm{SR}(\boldsymbol{\sigma}^{\eta})=\mathrm{IR}(\boldsymbol{A})$.

4 SUPPLEMENTARY NOTE 4: Conversion Rates Under LF_1

In this section, we show how the conversion rate, defined in the asymptotic limit, can be defined in terms of the one-shot success probability. The conversion rate defined by Eq. (11) of the main text can be straightforwardly computed using the law of large numbers for independent events. For completeness, we provide the following short argument. Since each local filtering can be performed independently, we have a binomial distribution for k successful events out of N trials, namely

$$p(k:N):=\binom{N}{k}p_{\mathrm{succ}}^{k}(1-p_{\mathrm{succ}})^{N-k}. \tag{8}$$

Denoting the random variable of the number of successes by X, we can compute its mean value $\mu(X)=Np_{\mathrm{succ}}$ and variance $\mathrm{var}(X)=Np_{\mathrm{succ}}(1-p_{\mathrm{succ}})$. Considering the average success per trial $\bar{X}:=X/N$, we have $\mu(\bar{X})=p_{\mathrm{succ}}$ and $\mathrm{var}(\bar{X})=Np_{\mathrm{succ}}(1-p_{\mathrm{succ}})/N^2=p_{\mathrm{succ}}(1-p_{\mathrm{succ}})/N$. This converges to the exact rate $r=p_{\mathrm{succ}}$ with zero variance in the limit $N\to\infty$.

5 SUPPLEMENTARY NOTE 5: Qubit Assemblage with Three Inputs and Two Outcomes

Here, we show how to use our formalism to recover the pure-qubit example by Nery *et al.*[4]. Consider the two-qubit state in the Schmidt form $|\psi\rangle=\sum_{i=1}^{2}\mu_i|ii\rangle$ with $1>\mu_2>\mu_1>0$ and $\mu_2^2+\mu_1^2=1$. The reduced state of $|\psi\rangle$ is $\tau=\mu_1^2|1\rangle\langle 1|+\mu_2^2|2\rangle\langle 2|$. We consider Alice's measurement assemblage $\boldsymbol{A}$ to be the Pauli X, and Z, namely, $A_{a|0}=1/2(\mathbb{I}+(-1)^aX)$ and $A_{a|1}=1/2(\mathbb{I}+(-1)^aZ)$, where a is the outcome of the Pauli observable. Using the property in Eq. (36) of the main text again, the initial assemblage can be expressed as $\sigma_{a|x}=\tau^{1/2}A_{a|x}\tau^{1/2}$ for all a and x. In this case, note that the SEO $\boldsymbol{B}$ is the same as the measurement assemblage $\boldsymbol{A}$. Inserting the SEO into the dual SDP formulations of IR in Eq. (31) of the main text, we can obtain a feasible solution of $\eta=\mathbb{I}/2$, which can be used to determine the optimal assemblage. We can now construct a local filter using Eq. (15) of the main text, as follows:

$$K:=\sqrt{\mu_1}(1/\mu_1|1\rangle\langle 1|+1/\mu_2|2\rangle\langle 2|). \tag{9}$$

The coefficient $\alpha=\sqrt{2\mu_1}$ can be obtained by Eq. (16) of the main text.One can apply the local filter to the initial assemblage using Eq. (19) of the main text, leading to the optimal assemblage $\boldsymbol{\sigma}^*=1/2\boldsymbol{A}^T$, which can be obtained by considering the maximally entangled state and the same measurement assemblage. Finally, by Eq. (12) of the main text,the rate is $r=\alpha^2=2\mu_1$.

We note that instead of considering Eq. (16) of the main text,we use the lower bound of the success probability in Eq. (23) of the main text to determine the coefficient α'. We can construct the other local filter $K'=\frac{\mu_1^2}{\mu_2^2}|1\rangle\langle 1|+|2\rangle\langle 2|$, which can also transmit the initial assemblage to the same optimal assemblage. However, in this case, the rate $r'=\alpha'^2=2\mu_1^2<r$. This construction is the same as that proposed by Nery *et al.*[4].

References

1. Gallego, R. & Aolita, L. Resource theory of steering. *Phys. Rev. X* **5**, 041008, DOI: 10.1103/PhysRevX.5.041008 (2015).
2. Ku, H.-Y. *et al.* Einstein-Podolsky-Rosen steering: Its geometric quantification and witness. *Phys. Rev. A* **97**, 022338, DOI: 10.1103/PhysRevA.97.022338 (2018).

3. Uola, R., Budroni, C., Gühne, O. & Pellonpää, J.-P. One-to-one mapping between steering and joint measurability problems. *Phys. Rev. Lett.* **115**, 230402, DOI: 10.1103/PhysRevLett.115.230402 (2015).
4. Nery, R. V. *et al.* Distillation of quantum steering. *Phys. Rev. Lett.* **124**, 120402, DOI: 10.1103/PhysRevLett.124.120402 (2020).
